# Potential proarrhythmic side effect of high dose and prolonged infusion of sodium nitroprusside through calcium ion reduction: a case report

**DOI:** 10.1093/ehjcr/ytad619

**Published:** 2023-12-18

**Authors:** Luca Fazzini, Mauro Gori, Christian Cadeddu Dessalvi, Michele Senni

**Affiliations:** Clinical Cardiology Unit, University Hospital of Cagliari, SS554, 4, 09042 Monserrato, Cagliari, Italy; Cardiovascular Department, ASST Papa Giovanni XXIII Bergamo, 24127 Bergamo, Italy; Clinical Cardiology Unit, University Hospital of Cagliari, SS554, 4, 09042 Monserrato, Cagliari, Italy; Cardiovascular Department, ASST Papa Giovanni XXIII Bergamo, 24127 Bergamo, Italy

**Keywords:** Advanced heart failure, Pharmacology, Sodium nitroprusside, Arrhythmias, Case report

## Abstract

**Background:**

Sodium nitroprusside (SNP) is an excellent drug in acute decompensated heart failure (HF) patients with high vascular peripheral resistance. Its prolonged administration may cause thiocyanate accumulation and toxicity. A proarrhythmic side effect has never been reported.

**Case summary:**

Herein, we report a case of an adult male affected by advanced HF due to a valvular cardiomyopathy admitted to our intensive cardiology unit with severe decompensation and waiting for a heart transplant. He was treated for several weeks with high-dose SNP, due to severe pulmonary hypertension and an extremely labile haemodynamic profile. He progressively developed high thiocyanate levels and, concomitantly, free calcium ion depletion, despite normal total calcium levels, with iterative ventricular arrhythmias. Calcium ion depletion was not responsive to calcium supplementation. We suspected a causative role of thiocyanate since the negatively charged sulfur atom of the thiocyanate molecules could bind the positively charged free calcium ions, leading to a free calcium ion depletion. Thus, we cautiously reduced SNP dosage, according to the patient's haemodynamic profile, with concomitant progressive free calcium ion normalization, thus reducing the arrhythmic burden of the patient, being able to finally perform heart transplantation.

**Conclusion:**

We describe for the first time a proarrhythmic side effect of prolonged SNP administration, namely, calcium ion depletion, likely related to thiocyanate toxicity. Despite aggressive calcium supplementation, the only way to reduce the arrhythmic burden was SNP down titration.

**Learning points**

- To recognize the potential drawback of prolonged infusion of sodium nitroprusside (SNP).
- To describe severe depletion of calcium ion as a consequence of SNP treatment.
- To understand that calcium supplementation is not useful and that the only effective treatment in such cases is down titration of SNP.

## Introduction

Patients with advanced heart failure (HF) in the acute phase of their disease may require inotropes, vasoactive drugs, and mechanical circulatory support.^1^ Acute HF is characterized by increased filling pressures, neurohormonal activation with arterial vasoconstriction, and, therefore, impairment of ventricular arterial coupling, as long as arterial elastance is disproportionally increased.^2^ Intravenous diuretics, vasodilators, and inotropes are the drugs of choice in this setting. Among intravenous vasodilators, sodium nitroprusside (SNP) has the peculiar characteristics of being able to reduce pulmonary pressure, which justifies its widespread use, in particular, to test pulmonary vasoreactivity and manage acute decompensation.

We report the case of a patient on the waiting list for heart transplantation with acute HF and reversible severe pulmonary hypertension who needed long-term SNP infusion and developed thiocyanate increase and, concomitantly, free calcium ion depletion with normal total calcium levels. This condition was associated with life-threatening arrhythmias.

## Case presentation

A 50-year-old male patient with a history of congenital aortic stenosis surgically corrected previously and with chronic HF due to severe left ventricular dysfunction presented to our emergency department complaining of dyspnoea. Due to acute decompensated HF with beginning cardiogenic shock, he was admitted to our intensive cardiology unit. Physical examination revealed pulmonary congestion with slightly cold extremities. The blood examinations showed normal renal and liver function, normal total and free calcium, 9.4 mg/dL (NV 8.5–10.2 mg/dL) and 1.16 mmol/L (NV 1.1–1.3 mmol/L), respectively, and lactates were mildly increased. The chest X-ray confirmed pulmonary congestion. On echocardiography, we found severe biventricular dysfunction (left ventricular ejection fraction 20%, right ventricular fractional area change 25%), moderate mitral regurgitation, and severe pulmonary hypertension (estimated pulmonary artery systolic pressure of 65 mmHg). Since the patient was unstable, adrenaline (0.05 µg/kg/min) and high-dose SNP (2.47 µg/kg/min) were started. Additionally, an intra-aortic balloon pump (IABP) was placed. Right heart catheterization with a Swan-Ganz catheter revealed severe pulmonary hypertension reversible with SNP and, therefore, the patient was inserted into the heart transplant list.

Patient haemodynamic was persistently unstable and we were able to avoid endotracheal intubation, with its inherent high infective burden, through continuous infusion of inotropes, prolonged IABP, and high dose of SNP (2.47 µg/kg/min). One of the side effects of SNP is thiocyanate toxicity. Thiocyanate levels were monitored and increased up to 10 mg/100 mL (NV < 3). An attempt to reduce the dose of SNP to 1.1 µg/kg/min failed due to acute pulmonary oedema rapidly reversed after the restoration of a higher SNP dose. However, after 3 weeks, the free calcium ion progressively decreased up to 0.94 mmol/L, despite normal total calcium and calcium supplementation intravenously and orally. Since the arrhythmic burden, characterized by frequent premature ventricular beats and iterative non-sustained monomorphic ventricular tachycardia, markedly increased, we started lidocaine which was not effective. The patient was not taking any antibiotics, albumin, or other drug capable of bonding the calcium ion; total plasmatic proteins were normal.

**Figure 1:**
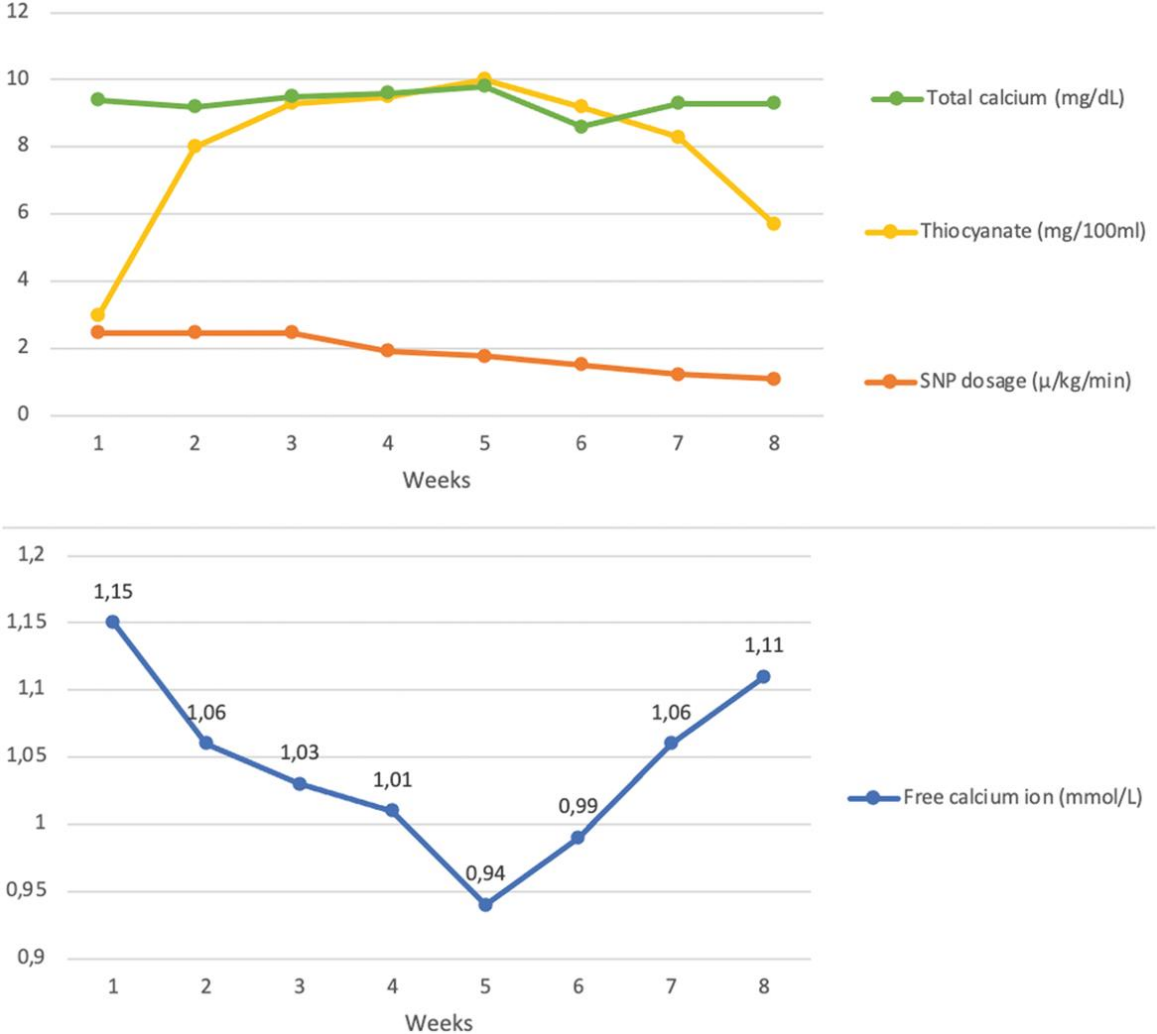
Graphical representation of total calcium levels, thiocyanate concentration, and sodium nitroprusside dosage during hospitalization weeks and concomitant free calcium ion levels variations.

Since we suspected a possible causative role of thiocyanate, which might bond the double positively charged free calcium ion with its two negatively charged sulfur atoms, explaining the normal total calcium level and the low calcium ion, we carefully reduced the SNP dose within several days. This allowed us to reduce thiocyanate to 6 mg/100 mL, without compromising patient haemodynamics. Concomitantly, the free calcium ion tended to normalize, reducing the arrhythmic burden (*Figure 1*).

## Discussion

Sodium nitroprusside combines arterial and venous vasodilation effects. The effect on the arterial side leads to a decreased afterload, better left ventricular ejection, mitral regurgitation reduction, and therefore stroke volume and cardiac output increase.^3^ On the other side, venodilation causes shifting from stressed to unstressed volume, preload, and central venous pressure reduction.^4^ The global effect is the reduction in left ventricular filling pressure and pulmonary capillary wedge pressure with increased cardiac output.

The most frightful side effects of SNP include hypotension and thiocyanate toxicity. However, even in patients with systolic blood pressure under 110 mmHg, only 5% of patients required SNP discontinuation due to hypotension.^5^ While hypotension is an immediate effect of SNP infusion, thiocyanate toxicity requires long-term continuous administration at high doses.

Sodium nitroprusside metabolism starts in red blood cells, where the interaction with the oxyhaemoglobin produces cyanide ions and methemoglobin. The cyanide ion has two pathways, it could bind the methemoglobin to generate cyanmethemoglobin or be metabolized to thiocyanate in the liver and kidney by the rhodanase enzyme. According to these metabolic pathways, SNP may cause toxicity by thiocyanate and cyanide accumulation. That is why SNP toxicity is related to prolonged administration or occurs in patients with liver or kidney failure and thiocyanate weekly dosage is recommended.^6,7^ While thiocyanate toxicity causes fatigue, tinnitus, seizures, mental status changes, and psychosis, cyanide toxicity might lead to coma, metabolic acidosis, and respiratory arrest. Interestingly, none of these well-recognized symptoms were present in our patient, despite the high thiocyanate levels.

An SNP proarrhythmic effect has never been described. Nonetheless, in our case, during prolonged SNP administration, the increased thiocyanate blood levels were associated with calcium ion depletion. The most likely explanation is that two negatively charged sulfur atoms of the thiocyanate molecules could bind the free calcium ions with a double positive charge, leading to a free calcium ion depletion assessed by blood gas analysis, with a normal total calcium level. The free calcium ion normal range is included between 1.16 and 1.29 mmol/L. Since the calcium ion is responsible for cell membrane homeostasis, its low level may impair the cytosolic concentrations leading to early and delayed afterdepolarization and enhanced automaticity further promoting a calcium-induced arrhythmic burden increase.

When the free calcium ion goes below 1.05 mmol/L and an increased ventricular arrhythmic burden is encountered, it is important to slowly reduce SNP dosage, which seems to be the only way to correct low calcium ion levels, since even intravenous gluconate calcium and oral carbonate calcium were not effective.

We describe for the first time a proarrhythmic side effect of prolonged SNP infusion, likely related to thiocyanate accumulation and free calcium ion binding, which, despite aggressive calcium supplementation, was normalized only after progressive and slow SNP down titration.

## Data Availability

All data are incorporated into the article.

## Lead author biography

I am a 27-year-old cardiologist resident in the University of Cagliari. Currently, I am attending the Cardiology Department at the ASST Papa Giovanni XXIII in Bergamo with Professor Michele Senni. I work in intensive cardiology care and I deal with heart failure. Moreover, I am attending a Master of ‘Intensive Cardiology Care’ at the University of Milan.
